# Marine and Freshwater Plants: Challenges and Expectations

**DOI:** 10.3389/fpls.2019.01545

**Published:** 2019-11-22

**Authors:** Eric Maréchal

**Affiliations:** Laboratoire de Physiologie Cellulaire et Végétale, UMR 5168 CNRS, CEA, INRAE, Université Grenoble Alpes, IRIG, CEA Grenoble, Grenoble, France

**Keywords:** algae, phytoplankton, seaweed, photosynthesis, endosymbiosis, bloom, holobiont, blue biotechnologies

## Abstract

The past decades have seen an increasing interest on the biology of photosynthetic species living in aquatic environments, including diverse organisms collectively called “algae.” If we consider the relative size of scientific communities, marine and freshwater plants have been overall less studied than terrestrial ones. The efforts put on land plants were motivated by agriculture and forestry, applications for human industry, easy access to terrestrial ecosystems, and convenient cultivation methods in fields or growth chambers. By contrast, the fragmentary knowledge on the biology of algae, the hope to find in this biodiversity inspiration for biotechnologies, and the emergency created by the environmental crisis affecting oceans, lakes, rivers, or melting glaciers, have stressed the importance to make up for lost time. Needed efforts embrace a broad spectrum of disciplines, from environmental and evolutionary sciences, to molecular and cell biology. In this multiscale view, functional genomics and ecophysiology occupy a pivotal position linking molecular and cellular analyses and ecosystem-level studies. Without pretending to be exhaustive and with few selected references, six grand challenges, requiring multidisciplinary approaches, are introduced below.

## Evolution, Taxonomy and Biodiversity of Photosynthetic Organisms in Aquatic Ecosystems

The biodiversity found in the depth of lakes, rivers, oceans, and even ice and snow has always been a source of wonder and curiosity. For centuries, species have been described with great morphological precision. Taxonomic views have been revised regularly and are still debated, now based on molecular, chemical, and ultrastructural traits (Adl et al., 2019). The very first challenge is to provide clarity, whenever possible, on the evolution and taxonomy of photosynthetic organisms and their close nonphotosynthetic relatives. This is not an easy task. Organisms simplistically called “algae” encompass clades in extremely distant branches in the Tree of Life. In particular, the evolution of eukaryotic algae is far from being established. This task depends on our understanding of the origin of prokaryotic and eukaryotic cells, and their evolution *via* series of endosymbioses and gene transfers. We have left the naïve age when a kingdom of “plants” opposed that of “animals,” when species were simply bounded by reproductive barriers. The same species can be considered as “algae,” “plants,” or “photosynthetic protists,” with reference to alternative taxonomic views. Nevertheless, we should aim to a consensus. A primordial event has been the engulfment of an ancestral cyanobacterium that gave rise to an intracellular organelle, limited by two membranes, called the chloroplast. Many chloroplast proteins are from noncyanobacterial origin, raising the question of the contribution of additional prokaryotic partners in its emergence (Cenci et al., 2017). This primary endosymbiosis event is at the basis of Archaeplastida, who radiated in three lineages, identified by their photosynthetic pigments: the green lineage, comprising green algae and embryophytes forming together the Viridiplanta, the red lineage, consisting of red algae, and the glaucophytes, comprising few species (Marechal, 2018). In addition, a more recent cyanobacterial endosymbiosis has occurred in Paullinellida, at the origin of a photosynthetic organelle called the chromatophore (Archibald, 2017). The vast majority of photosynthetic eukaryotes populating aquatic ecosystems are neither Archaeplastida nor Paullinellida, as they derive from secondary endosymbiosis events that have occurred later, after the engulfment and reduction of a green or a red alga inside a secondary eukaryotic host cell (De Vargas et al., 2015). Some organelles have also been “stolen” from one cell to another by a process known as kleptoplasty (Hehenberger et al., 2019). The green and red lineages can, therefore, be traced in secondary endosymbionts, for instance in Euglenida and Heterokonta, respectively. In all cases, massive endosymbiotic and horizontal gene transfers have occurred, indicating the contribution of multiple prokaryotes, eukaryotes and possibly viruses in the emergence of novel clades. Genomes have a mosaic organization resulting from this complex history (Obornik, 2018). Major phyla of photosynthetic eukaryotes have nonphotosynthetic, and even plastid-free relatives. In the red lineage, *Chromera velia* is close to Apicomplexa, such as the malaria causative agent *Plasmodium falciparum*, containing a nongreen plastid. In the green lineage, *Euglena gracilis* is close to human parasites such as *Trypanosoma cruzi*, the causative agent of Chagas disease, devoid of any plastid. Although a rough estimate of more than two thirds of the biodiversity of photosynthetic eukaryotes derive from a secondary endosymbiosis (De Vargas et al., 2015), Euglenophyta, Chlorarachniophyta, Cryptophyta, Haptophyta, Heterokontophyta, Dinophyta, etc., have been far less studied than green algae and vascular plants. A fascinating but really challenging task is, therefore, to trace, reconstruct and refine missing events. The mosaic architecture of genomes needs to be characterized with care. The definition and positioning of clades in the Tree of Life and the refining of taxonomy are also crucial for large-scale studies at ecosystems’ level. Environmental DNA and/or RNA, analyzed with barcoding, meta-genomic, and/or meta-transcriptomic methods, are used to define “operational taxonomic units,” or OTUs (De Vargas et al., 2015). Better characterizations of species and their mosaic genomes are therefore necessary to improve the assignment and interpretation of OTUs, and address the structure and dynamics of natural populations and communities in water ecosystems.

## Propagation of Unicellular, Colonial and Multicellular Organisms, Ecotypes and Cryptic Species, Life Cycles, Genetic Diversification and Adaptation Mechanisms

Schematically, cell and organism propagation is less constrained in water. Simple cell division is actually the basis of cyanobacteria propagation in all environments, developing resisting forms, spores, and cysts to cope with adverse periods. In water, mitotic divisions of phytoplankton, in haploid or diploid states, can be sufficient to populate large biogeographic areas. By contrast, a fixed lifestyle marks some major clades of macroalgae, most spectacular being kelp forests. Although vegetative reproduction occurs in land plants as well, breeding is a major process for the expansion of terrestrial species and it coincides with the acquisition of genetic variations. In aquatic ecosystems, it seems that all reproductive processes exist and allow genetic diversification. Sexual reproduction of some eukaryotic algae may be minor, undemonstrated or even absent, leading to the possible existence of cryptic species (Grimsley et al., 2010). Genetic variations can be acquired by a multitude of processes other than recombination during meiosis. These mechanisms include spontaneous mutations, permanent exposure to free or viral DNA in the water environment, and numerous mechanisms of horizontal gene transfers. The importance of transposable elements needs to be evaluated in aquatic photosynthetic organisms, compared to the role they have as drivers of genome evolution in numerous terrestrial plants. Emergence of allopolyploidy, combining parental genomes from distinct species, has been little described in marine algae, with noticeable exceptions such as the hybrid diatom *Fistulifera solaris* (Nomaguchi et al., 2018). Due to the lack of epigenetic reprogramming during meiosis, transgenerational effects are particularly long-lived and relevant in asexually reproducing organisms. Transgenerational plasticity may be mediated through various nongenetic processes including parental effects (e.g., transmission of nutrients, hormones, proteins, and mRNA) by epigenetic changes (e.g., DNA methylation, histone modification, and noncoding small RNAs). Vegetative reproduction is also a key to the existence of longer generations in life cycles of unparalleled variety and complexity. The alternation of diploid and haploid generations and sex determination can follow schemes that are often very sophisticated and unique to some algal and protist phyla (Umen and Coelho, 2019). A second challenge is, therefore, to combine our efforts to characterize the diverse and complex life cycles of marine and freshwater plants. Molecular and cellular mechanisms of mitosis and meiosis, genetic determinants of sexual dimorphism in haploid and/or diploid generations, formation of mononucleated or polynucleated cells, acquisition of multicellularity, differentiation of cell types and gametes, spores and cysts, etc., need to be characterized in all major clades of unicellular and colonial algae, photosynthetic protists and eventually seaweeds.

## Study Models

Efforts on the development of the *Chlamydomonas* model have been remarkable, as a large indexed, mapped mutant library has been developed enabling reverse genetics and genomic phenotyping (Li et al., 2016) and it will be extremely useful to address biological questions for green algae. Such phenotyping allowed for instance the identification of new genes required for oxygenic photosynthesis (Li et al., 2019). Nevertheless, the complexity of the intracellular compartmentalization deriving from secondary endosymbiosis, mentioned above, makes the cells of *Chlamydomonas*, *Arabidopsis*, yeast or any kind of popular animal cell model, frustratingly simple. Sequenced genomes and metagenomes reveal lists of genes with no homolog, or with homology restricted to some clades. A third challenge is to accept that we need to perform molecular and cellular dissections of unknown gene products, unknown subcellular structures, using the tools of cell fractionations and biochemistry widely available in the 1990’s, one thought we would not need following the release of complete genome sequences of dominating study models. Fortunately, analytical methods for the determination of the proteome, lipidome, metabolome, etc. of isolated cells and cell fractions have a sensitivity requesting much lower amounts of starting material [e.g., (Lupette et al., 2019)]. Strategies can also help addressing the role of unknown genes, circumventing the biochemical characterization of cell extracts, by attempting to interpret gene expression levels in some physiological states or in response to environmental conditions, and by the use of genetic engineering and editing methods [e.g., (Kroth et al., 2018; Stukenberg et al., 2018)]. The challenge is then to develop sufficiently stable reference cell lines, with available sequence data and appropriate genetic transformation methods. Established model species include, and are not limited to *Synechocystis* spp., *Chlamydomonas reinhardtii*, *Ostreococcus tauri*, *Cyanidioschyzon merolae*, *Euglena gracilis*, *Thalassiosira pseudonana, Phaeodactylum tricornutum*, *Nannochloropsis* spp., *Guillardia theta*, *Bigelowiella natans*, *Chondrus crispus*, *Ectocarpus siliculosus*, etc. (Gachon et al., 2007). A monocot, *Spirodela polyrhiza*, needs also to be added to this list, to comprehend angiosperm adaptation to aquatic habitats (Wang et al., 2014; Xu et al., 2019). Each model is key to address questions in relation with specific clades of algae and plants. None of them is perfect, but they have become more or less popular and distributed in different laboratories. Novel model or nonmodel species are also critical to address specific or novel questions, such as *Galdieria sulphuraria*, to study life in sulfuric acidic hot springs (Hirooka and Miyagishima, 2016), *Chromera velia or Vitrella brassicaformis* to address the origin of Apicomplexa parasites, etc. (Fussy et al., 2019). Efforts should, therefore, be focused on well-selected organisms. The recent methodic explorations of aquatic ecosystems with new technologies, combining environmental DNA analysis (Carradec et al., 2018) with the collection of live samples may help us identifying novel study models. Specific tools for molecular engineering (vectors, transformation methods, RNA interference, genetic recombination, overexpression, etc.), gene editing (e.g., TALEN, Crispr-Cas9, etc.) and phenotype characterization (high-resolution imaging, single-cell analyses, etc.) need to be developed for these cell lines, sometimes difficult to genetically transform.

## Diversity of Unicellular and Multicellular Architectures, Cell Compartments, Photosynthetic Processes and Metabolic Pathways, Genetic Regulation Systems, Signaling and Developmental Processes

The most obvious gaps of knowledge concern the complexity of functional architectures, found for instance in “exotic” cell structures (e.g., nucleomorph in Cryptophyta or Chlorarachniophyta, mineralized cell wall in Coccolitophores or Diatoms, chromatophore in Paullinelidae, ocelloid in some Dinoflagellates). The organization and origin of the secondary or complex plastid in Chromista, bounded by four membranes [e.g., (Flori et al., 2016; Cavalier-Smith, 2018)] is an essential question that needs to be addressed. Multicellularity acquired by fixed macroalgae, living in a vertically contrasted environment in terms of light, salinity, exposure to the air at low tides, etc (Smale, 2019) also seems to be guided by constrains differing from those found in terrestrial ecosystems, in which the acquisition of vascular tissues proved critical to link organs developing in the soil and in the air. Photosynthesis and carbon metabolism, which seem to unify photosynthetic taxa, combine in fact common and distinct machineries and pathways that need to be unraveled [e.g., (Giovagnetti and Ruban, 2018)]. Pigmented photosystems, photoprotection mechanisms, CO_2_ concentration and capture systems, etc. need to be structurally and functionally deciphered. The biology of the pyrenoid, the relation between autotrophy, heterotrophy, and mixotrophy, are critical questions. Concerning development and differentiation, the mechanisms controlling gene expression, by the action of specific transcription factors but also *via* poorly characterized epigenetic mechanisms, controlling gene regulatory networks, need to be addressed (Tirichine and Bowler, 2011). The fourth grand challenge we face is, therefore, to dissect and clarify functional organizations that are unique to clades of marine and freshwater plants, in relation to their environment.

## Abiotic and Biotic Interactions, Role in Geochemical Cycles, Population and Community Dynamics, Holobionts, Evolution in the Context of Global Change

Predominance of water is not sufficient to characterize an aquatic ecosystem. Habitats can be marked by spatiotemporal variations in light intensity and chromatic quality, temperature, pH, concentrations of salts, of all kinds of nutrients such as nitrogen, phosphorus, sulfur, iron, silica, etc., of CO_2_, noxious gases, particles, pollutants, etc. Water can also have various states, from liquid to snow, ice and even droplets in aerosols. Connectivity between water habitats can provoke a brutal transfer from a physicochemical environment to another, such as in estuaries, mangroves, upwelling, or any kind of displacement in a water column, during freezing and melting. Environmental changes can also be progressive between highly contrasted conditions. For instance, in subpolar areas, photosynthetic organisms are exposed to light during a few months, whereas the rest of the year consists in a long night. Connectivity with other habitats, such as sediments, soils, and air is also common. Interactions with the abiotic environment need therefore to be established in a broad variety of conditions, including various transitional gradients between highly contrasted conditions. Intraspecies and interspecies biotic interactions need also to be addressed, providing clues on the dynamics of populations and communities and helping to characterize natural associations of microbial species, or holobionts [e.g., (Lachnit et al., 2015; Arnaud-Haond et al., 2017; Decelle et al., 2019; Ziegler et al., 2019)]. These include, and are not limited to, interactions with bacteria, grazers, viruses, the formation of photosymbiotic and/or parasitic associations. This fifth challenge, aiming at characterizing abiotic and biotic interactions, is based on molecular mechanisms, including intracellular and intercellular signaling molecules (e.g., calcium, cyclic nucleotides, oxylipins, phosphoinositides, “infochemicals,” “phytohormones,” etc.), their receptors, the signaling cascades they trigger and responses from genetic reprogramming to metabolic remodeling, activation of immune mechanisms and cell differentiation. Gained knowledge will be essential to improve our understanding of the role(s) of marine and freshwater plants in geochemical cycles and trophic networks. Knowledge is also critically needed to address the proliferation of algae in phytoplankton blooms, including harmful cyanobacterial blooms (Alvarenga et al., 2017), and in seaweed blooms, such as green tides occurring with an increasing frequencies on coastal areas (Zhao et al., 2019), or sargassum blooms that are so huge that they are considered to form the so-called Great Atlantic Sargassum Belt (Wang et al., 2019). Photosynthetic algae benefit from increased CO_2_ availability and their proliferation can be considered as a marker of global climate change. In the case of blooms of green algae in the snow cover, forming so-called “red snow” in polar area and high altitudes, the pigmentation increases the albedo and accelerates melting: algae are then actors of climate change by a positive feedback loop (Kintisch, 2017). Understanding abiotic and biotic relations is, therefore, necessary to address the impact on, and the role of marine and freshwater plants in the environmental crisis.

## Expectations for the Development of Algae-Based Technologies

Last and not least, aquatic photosynthetic organisms are seen as a still to be explored mine of biomolecules, such as lipids, carbohydrates, pigments, secondary metabolites, etc. for high-value applications, including feed, food, health or cosmetics. They are also a promising feedstock for green chemistry (sometimes called blue chemistry), biomaterials and bioenergies (Scaife and Smith, 2016; Lupette and Maréchal, 2018). Algae can also serve as cell factories after genetic engineering. Expectations are high, but numerous biological and technological issues need to be resolved. Some species have reached the status of industrial production strains. Efforts are put to characterize and improve the domestication of microalgae such as *Spirulina* spp., *Chlorella* spp., *Haematococcus pluvialis*, *Nannochloropsis* spp., nonphotosynthetic Thraustochytrids, and seaweeds and kelp, such as *Saccharina japonica*, *Undaria pinnatifida*, *Porphyra tenebra*, etc. Search for valuable novel strains is critical. In this last challenge, it seems that two major obstacles need to be overcome at the level of the developed strains. The first one is the production of biomass that seems to depend on the limitations of photosynthesis efficiency. The second obstacle is to succeed controlling carbon partitioning in cultivated strain to produce valuable biomolecules, for instance oil, with an economically viable yield and quality. Other key questions include the development of appropriate cultivation systems, nutrient supplies complying with sustainability models, and low-energy harvesting and extracting methods. All these applied issues rely on the advance of fundamental research.

In conclusion, the field of marine and freshwater plant science is immense and fascinating. The advancement of knowledge should benefit from cross-fertilization of disciplines, from environmental sciences to molecular and cell biology, from the biophysics of photosynthesis and biochemistry of metabolism to biotechnological developments. No doubt that this field will lead to important discoveries changing our views on eukaryotes’ biodiversity and ecosystems and leading to the development of an alga-based economy.

## Author Contributions

As the sole author, this manuscript was fully handled by Dr. EM.

## Funding

The author is supported by the Centre National de la Recherche Scientifique, the Commissariat à l’Energie Atomique et aux Energies Alternatives, the Institut National de Recherche pour l’Agriculture, l’Alimentation et l’Environnement, the Université Grenoble Alpes, and by grants from the French National Research Agency (Oceanomics ANR-11-BTBR-0008, GlycoAlps ANR-15-IDEX-02, GRAL Labex ANR-10-LABEX-04, and EUR CBS ANR-17-EURE-0003).

## Conflict of Interest

The author declares that the research was conducted in the absence of any commercial or financial relationships that could be construed as a potential conflict of interest.
